# Long-lasting insecticide-treated bed net ownership, utilization and associated factors among school-age children in Dara Mallo and Uba Debretsehay districts, Southern Ethiopia

**DOI:** 10.1186/s12936-020-03437-9

**Published:** 2020-10-15

**Authors:** Zerihun Zerdo, Hilde Bastiaens, Sibyl Anthierens, Fekadu Massebo, Matewos Masne, Gelila Biresaw, Misgun Shewangizaw, Abayneh Tunje, Yilma Chisha, Tsegaye Yohannes, Jean-Pierre Van Geertruyden

**Affiliations:** 1 Department of Medical Laboratory Science, College of Medicine and Health Sciences, Arba Minch University, Arba Minch, Ethiopia; 2 Global Health Institute, Antwerp University, Antwerp, Belgium; 3 Department of Family Medicine and Population Health, Antwerp University, Antwerp, Belgium; 4 Department of Biology, College of Natural Sciences, Arba Minch University, Arba Minch, Ethiopia; 5 Department of Public Health, College of Medicine and Health Sciences, Arba Minch University, Arba Minch, Ethiopia

**Keywords:** Llins ownership, Utilization, Associated factors, School-age children, Southern Ethiopia

## Abstract

**Background:**

Malaria is one of the major causes of morbidity and mortality among school-age children (SAC) in sub-Saharan Africa. SAC account for more than 60% of the reservoir of malaria transmission, but they are given less emphasis in prioritizing malaria prevention interventions. This study was aimed at assessing the ownership of long-lasting insecticide treated bed nets (LLINs), its utilization and factors associated with ownership of LLINs by households and LLINs utilization among SAC in malaria-prone areas of Dara Mallo and Uba Debretsehay districts in Southern Ethiopia, October to December 2019.

**Methods:**

This study is part of a baseline assessment in a cluster-randomized controlled trial. The data was collected through interview and observation, following a structured questionnaire, of 2261 SAC households. Univariable and multivariable multilevel logistic regressions were used to assess the association between LLINs ownership and utilization and potential predictor variables. Odds ratio (OR) and corresponding 95% confidence interval (CI) were used to determine the strength and statistical significance of association.

**Results:**

The ownership of at least one LLIN by households of SAC was about 19.3% (95% CI 17.7–21.0%) but only 10.3% % (95% CI 7.7–13.7%) of these households had adequate access of bed nets to the household members. Ownership of bed net was marginally affected by living in semi-urban area (adjusted OR = 2.6; 95% CI 1.0–6.9) and occupational status of the household head being a civil servant (adjusted OR = 2.7; 95% CI 0.9–7.9). About 7.8% (95% CI 6.7–10.0%) of all SAC participated in the study and 40.4% (95% CI 57.4–66.7%) of children in households owning at least one LLIN passed the previous night under LLIN. LLIN utilization by SAC conditional to presence of at least one net in the household was significantly correlated with education level of mother above grade 6 (adjusted OR = 3.4; 95% CI 1.3–9.3) and the household size to bed net ratio less than or equal to 2 (adjusted OR = 20.7; 95% CI 4.7–132.5).

**Conclusion:**

Ownership of bed net was lower than universal coverage of at least one bed net for two individuals. It is important to monitor replacement needs and educate mothers with low education level with their SAC on the benefit of consistent utilization of bed nets.

## Background

Malaria is one of the leading causes of morbidity and mortality among infectious diseases in the world [1]. It is caused by a protozoan parasite of the genus *Plasmodium* and transmitted through the bite of the female *Anopheles* mosquito. There are five known species of *Plasmodium* that cause malaria in humans: *Plasmodium falciparum, Plasmodium vivax, Plasmodium ovale, Plasmodium malariae*, and *Plasmodium knowlesi* [2]. An estimated 228 million malaria cases and 405,000 malaria deaths occurred in the world in 2018. Of these, 93% of cases and 94% of deaths due to malaria occurred in the African region [3].

There has been substantial decrease in the prevalence of malaria globally and also in the African region in the last two decades. Between 2010 and 2015, malaria incidence rates (new malaria cases) fell by 21% and the malaria mortality rate by 31% in the African region [4]. However, the rate of change in decline of malaria incidence remained 57 per 1000 at-risk population per year from 2014 to 2018 [3].

Malaria is seasonal in most parts of Ethiopia with the highest transmission season between September and October following the main rainy season from June to August [4, 5]. A study carried out in North Gonder zone revealed that the highest incidence of malaria was observed between June and November while the lowest transmission period was from December to early April [6]. The number of malaria cases in September, October, November, May, June, and July were above the average malaria cases in Boricha district in Southern Ethiopia, while those in March, January, February, December, August, and April were below average [7].

The National Malaria Control Programme (NMCP) deployed by the Ethiopian government and partners has led to a promising decrease in the burden of malaria. As indicated by data collected from 41 hospitals in Ethiopia, the overall malaria inpatient cases were 54% lower and malaria deaths were 68% lower in 2011 than that predicted by the trends during 2001–2005 [8]. Moreover, in 2015, deaths due to malaria decreased by 40% compared to the number of deaths due to malaria in 2005 [9]. Decreased exposure of children under the age of 5 years resulted in delayed development of immunity in their growth and nowadays school-aged children (SAC) have become a highly susceptible group of people [10–12]. Stratified analysis of studies targeting the general population, as well as studies focused on SAC, revealed that the burden of malaria was higher among this group of the population in Ethiopia [13–15]. The consequence of malaria infection on children affects their physical growth, mental development and overall economic development of the country [16, 17]. Above all, they were responsible for about 60% of infection to the mosquito vector for the transmission of the disease in all seasons [18].

The long-lasting insecticide-treated net (LLIN) is the cornerstone of malaria prevention in sub-Saharan Africa (SSA). Its effectiveness was dependent on universal coverage as well as consistent utilization [4]. Utilization of LLINs reduces the clinical attack of malaria, *Plasmodium* infection and death due to malaria. It reduced child mortality of all causes by 17%, which corresponds to 5.6 lives each year for every 1000 children protected compared to those who did not use bed nets. In addition, it reduced incidence of uncomplicated episodes of *falciparum *malaria by almost half [19].

In Ethiopia, LLIN planning is based on the WHO recommendation of one net for 1.8 people. The nets are distributed through rolling mass campaign every 3 years [20]. National coverage of bed net ownership and utilization conditional to ownership in Ethiopia were 40% and 61%, respectively [13]. However, a cross-sectional survey conducted in different parts of the country targeting different population segments indicated that coverage and utilization differ widely. In Jima, for example, overall ownership and utilization were 70.9% and 38.4%, respectively, in the community [21]. Near Gilgel Gibe hydroelectric power project, 56.6% of the households owned at least one bed net and of those, 60% slept under bed nets on the night preceding the survey [22]. In Shashogo district in southern Ethiopia, ownership and utilization of bed nets was very low. Only 15.8% of pregnant women’s households owned a bed net and half of those owning bed nets utilized them [23]. However, the coverage and utilization were high in Mirab Abay district in Gamo Gofa zone with, respectively, 89.9% and 85.5% [24].

There are few studies that assessed the utilization of bed nets by SAC. In studies comparing bed-net utilization between SAC and other population segments, SAC less likely used bed nets [16, 17]. Despite SAC becoming at high risk of malaria and malaria-associated morbidities, their access to and benefit from existing malaria prevention interventions is not well addressed. Previous studies mainly focused on assessing access to malaria prevention interventions among pregnant women and children under 5 years old. LLINs are one of the major malaria prevention interventions in use in SSA. Assessing coverage of bed-net ownership and utilization by households of SAC, and their associated risk factors are important for optimizing malaria responses in Ethiopia and similar settings in SSA in light of targets set in for global technical strategy of malaria, 2016–2030. This study was conducted in Uba Debretsehay and Dara Mallo districts in the former Gamo Gofa Zone. These districts were selected based on the burden of malaria contribution to the total malaria cases in the Zones. Based on the recent annual report of the districts health offices in June 2020, there were a total of 8391 confirmed malaria cases out of a total of 63,953 people residing in malarious area in Uba Debretsehay district while the respective numbers in Dara Mallo district were 7666 and 38,043. A total of 14,440 bed nets which is equivalent to 96% coverage in Dara Mallo district and 32,500 bed nets that is equivalent to 100% coverage were distributed in June 2017.

## Methods

### Study setting

**Fig. 1:**
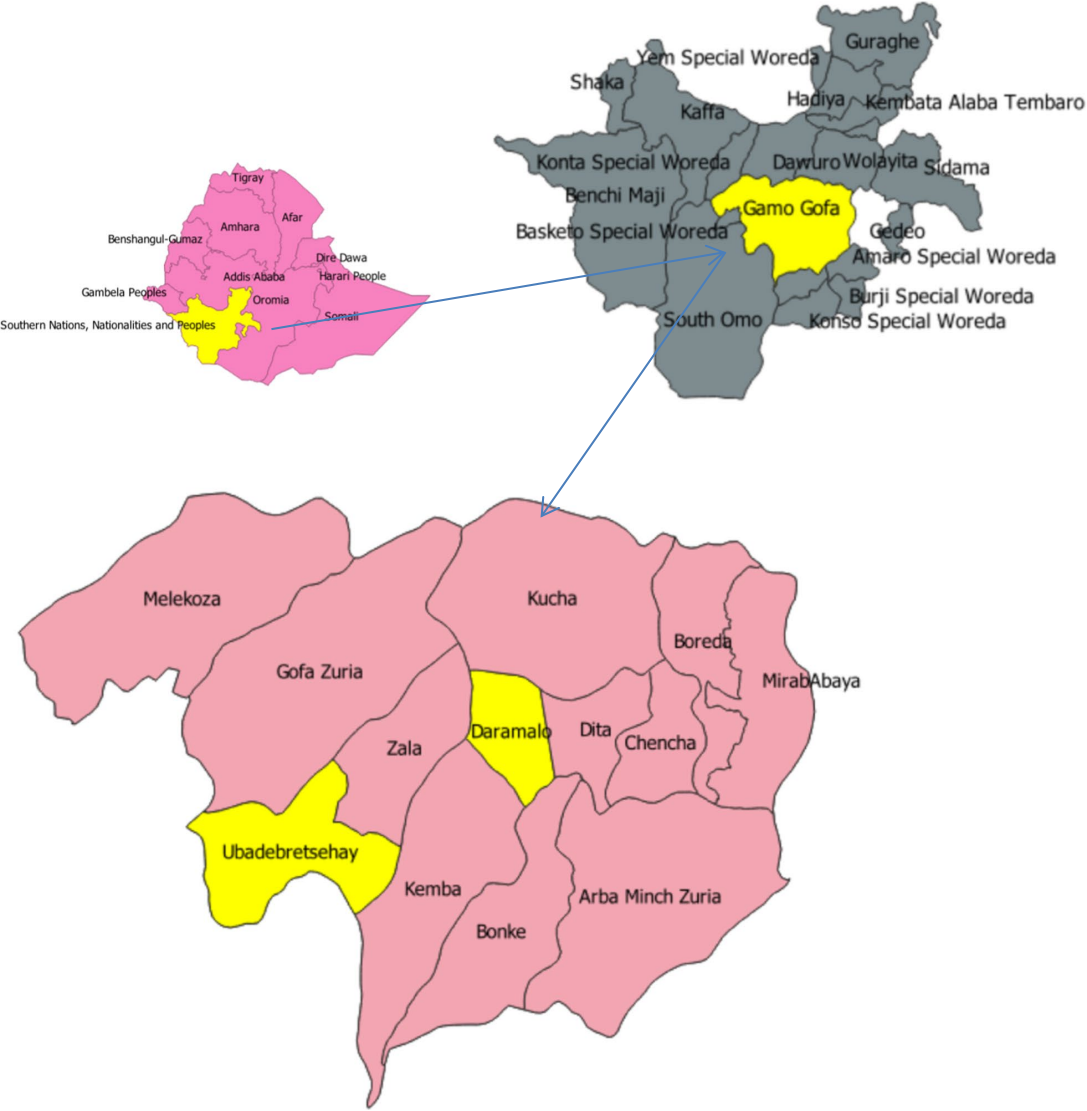
Location map of two districts selected from former Gamo Gofa zone, in Southern Nations, Nationalities and peoples region, Ethiopia

The study was conducted in Dara Mallo and Uba Debretsehay districts in Gamo and Gofa Zones. Both zones are found in Southern Nations, Nationalities and Peoples Region, which is the third populous region in Ethiopia. These districts are found in the western part of Arba Minch town, which is the capital of the former Gamo Gofa Zone (Fig. 1). Based on the 2007 national census, 150,145 people were living in the two districts, of whom 76,550 (51%) were male [25]. According to the recent update of the population by the respective districts, there were a total of 94,396 people in Uba Debretsehay district and 110,207 people in Dara Mallo district.

### Study design and sample size

A cross-sectional study was undertaken by recruiting 2261 children from 32 schools in the study area. The number of participants involved in this study was about 98.1% of the estimated sample size. The sample size estimated was 2304 SAC and their households. The estimated sample size is used to evaluate the effect of malaria prevention education on malaria, anaemia and cognitive development among SAC in the study area. This sample size was estimated by using formula recommended to calculate sample size for cluster randomized controlled trials [26, 27] for each of the two parallel arms. The assumptions used were 11.04% and 18.4% prevalence of malaria with and without the intervention, respectively; 95% level of confidence, 80% power, 72 cluster size and 0.025 intraclass correlation coefficient (ICC) with the calculated design effect of 2.775 and 15% loss to follow-up or non-response rate.

### Sampling techniques and data collection

A total of 3204 children attending primary education were approached at 32 primary schools. It had been planned to recruit 32 schools by using simple random sampling technique, but only 32 primary schools in malarious areas in the two districts could be recruited. Seventy-two children from grade 1 to 3 were selected using systematic random sampling technique from eligible children in each section of the students with a class roster as the sampling frame. All participants involved in the trial were included to address the research objective. The number of participants in the trial from each grade level (grade 1 to 3) was determined by the relative contribution of each grade level to the total enrolment of students in each school. Children who were attending their education in the schools during the data collection period were included in the study while those mentally not fit to respond to questions directed to them and with physical problems to measure their height were excluded from the study. Children enrolled to the study were used to trace their households. Participants’ households were approached by trained data collectors for interview. A pretested, structured questionnaire was used to collect data on demographics, water source, toilet structure, household assets and bed nets. The questions were adapted to local context from the Demographic and Health Surveys (DHS) malaria indicator survey household questionnaire [28]. The questionnaire was uploaded to tablets in Open Data Kit (ODK) data collection tool. The data collectors were trained on how to use the data collection tool and ethical procedures. They interviewed mothers or caregivers in conditions when the selected child mother was not alive, observed the toilet structure and the number and position (hung over sleeping place or not) of bed nets in the households.

Four indicators of malaria prevention measures by bed net utilization in the current study were used, but ownership and utilization of bed nets by SAC conditional to presence of at least one bed net in the household were dependent variables.

Indicator I: ownership, which is defined as SAC households those had at least one LLIN among SAC households participated in the survey.

Indicator II: utilization, which is defined as proportion of SAC passed the previous night under LLINs irrespective of ownership of LLINs.

Indicator III: conditional utilization, which is defined as proportion of SAC passed the previous night under LLINs conditional to presence of at least one LLIN in the household.

Indicator IV: access, which is defined as proportion of SAC households those had at least one bed nets for two household members among those owning at least one LLINs.

Indicator V: utilization conditional to access, which is defined as proportion of SAC passed the previous night under LLINs conditional to bed net access in the household [29].

In addition to SAC related indicators, any person from the household passed the previous night under bed net irrespective of presence or conditional to presence of at least one LLIN in the household were also assessed.

Indicator VI: any membered utilized, which is defined as any member of SAC household that passed the previous night under bed net among all surveyed SAC households.

Indicator VIII: conditional any membered utilized, which is defined as any member of SAC household that passed the previous night under bed net conditional to presence of at least one LLIN in the household.

### Data analysis

The data collected by using ODK data collection tool with tablet apparatus were converted to Microsoft Excel using ODK Briefcase. These data are imported to RStudio version 4.0.0 statistical software and STATA version 14 to predict the principal components (PCA). The variables used in predicting the wealth index of the household were the type of toilet used by the household; the source of drinking water for the household; the presence of sponge mattress in the household, the floor of which the house is constructed; the outer cover of the house, the total number of milk cows in the house; the number of other cows in the house; the number of goats and the number of poultry in the households. The predicted index from PCA is categorized into 4 quartiles to determine the wealth index of the households. Cleaned data were subjected to descriptive statistical analysis, such as proportion for categorical variables and plots and descriptive measures for continuous data. The survey data used in this study indicate a hierarchical nature in which children included were nested within the schools. Children within the same school will be similar to each other than students in another school. Thus, mixed effects multilevel logistic regression with random intercepts by schools for the outcome variables was used to assess the individual level fixed effect of correlates on the outcome variables. In the analysis of factors influencing ownership and use of bed net glmer command in lme4 R package was used. In the analysis of factors influencing bed net utilization, children with at least one bed net in their household were included. Odds ratio (OR) and corresponding 95% level of confidence (CI) were used to assess the strength of association between bed-net ownership or utilization and potential predictor variables. Variables that are significantly associated with bed net ownership and utilization in univarite multilevel logistic regression were taken to multivariate multilevel logistic regression. The fit of the mode in predicting the outcome variables were checked by Akaike Information Criterion (AIC). The averages of the household coordinates taken by global positioning system (GPS) were used to represent the coordinates of each school. The geospatial data was added to Quantum Geographical Information System (QGIS) to present the ownership and utilization percentages at school level.

### Ethical consideration

The trial was approved with written consent procedure to be followed by the Institutional Research Ethics review Board (IRB) in College of Medicine and Health Sciences, Arba Minch University with the reference number of IRB/154/12. Official permission letter to conduct the research was submitted to district health offices and education offices in Dara Mallo and Uba Debretsehay districts. Support letters written by the respective education offices were given to each participating school and written consent was obtained from school head teachers. Parents of the selected SAC were invited to come to the schools and written consent was obtained before data collection at school and household level, which was documented in College of Medicine and Health Sciences, Arba Minch University.

## Results

A total of 2261 SAC and their households participated in this study: 1419 from Uba Debretsehay and 842 from Dara Mallo districts. Of participating SAC, 50.3% were female and 85.5% were from rural area. The mean age of children was 8.7 years with standard deviation (SD) of 1.6 years. Some 39.1, 28.1 and 32.8% of the children were attending education in grade 1, 2 and 3, respectively. The mean household size was 7.0 with the standard deviation of 2.0. The detailed socio-demographic characteristics of the participated SAC households were presented in Table 1.

**Table 1:** Socio-demographic characteristics of participants involved in Dara Mallo and Uba Debretsehay districts, Southern Ethiopia, 2019

Factor	Value categories	Frequency (%)
Place of residence	Rural	1928 (85.3)
	Semi-urban	185 (8.2)
	Urban	148 (6.5)
Gender of household head	Male	2103 (93.0)
	Female	158 (7.0)
Age of household head	≤ 34	584 (25.8)
	35–49	1501 (66.4)
	≥ 50	176 (7.8)
Occupation of household head	Farmer	1852 (81.9)
	Civil servant	158 (7.0)
	Merchant	115 (5.1)
	Housewife	72 (3.2)
	Daily laborer	31 (1.4)
	Others	33 (1.5)
Educational status of household head	Illiterate	1311 (58.0)
	Literate	949 (42.0)
Ethnicity	Gofa	1156 (51.1)
	Gamo	928 (41.0)
	Amhara	31 (1.4)
	Others	146 (6.5)
Age of child’s mother (caretaker)	≤ 34	1472 (65.1)
	35–49	751 (33.2)
	≥ 50	38 (1.7)
Occupation of child’s mother (caretaker)	Housewife	1867 (82.6)
	Farmer	189 (8.4)
	Merchant	89 (3.9)
	Employee	82 (3.6)
	Others	34 (1.5)
Educational status of mother (caretaker)	Illiterate	1682 (74.4)
	Literate	579 (25.6)
Gender of SAC	Female	1137 (50.3)
	Male	1123 (49.7)
Grade of SAC	Grade 1	884 (39.1)
	Grade 2	635 (28.1)
	Grade 3	740 (32.8)
Household size	Less than or equal to 5	457 (20.2)
	Greater than 5	1804 (79.8)

### Ownership of LLINs

**Fig. 2:**
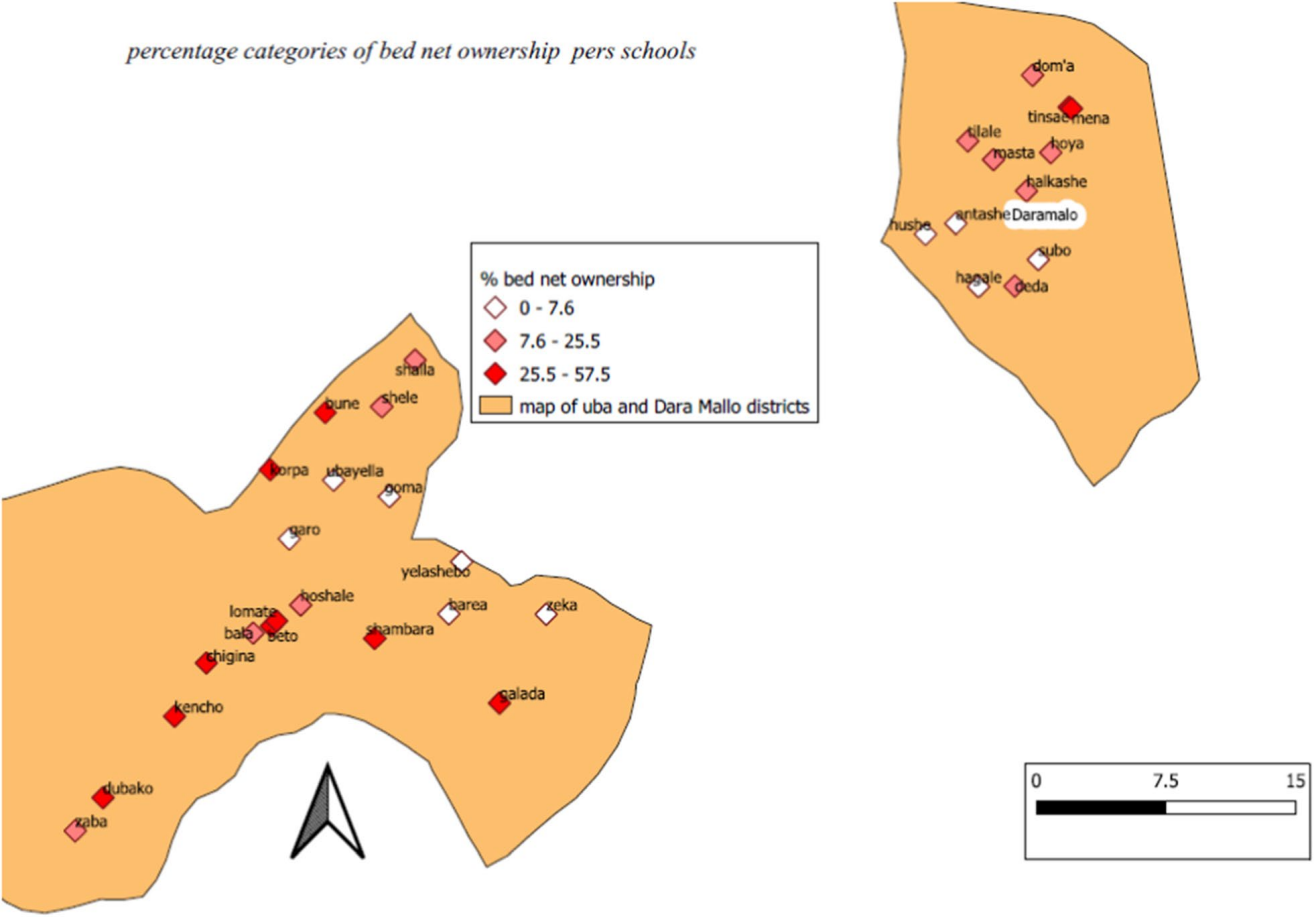
Percentage of bed net ownership among households per schools where participated children attend their education

Ownership of at least one LLIN at household level was 19.3% with 95% CI of 17.7–21.0%. The mean number of bed nets in a household owning bed nets was 1.8 (SD = 0.9), while 45 of the participated SAC households had adequate numbers of bed nets (at least one bed net for 2 household members). Overall ownership of at least one bed net and adequate access per district were indicated in Table2. There was wide difference in ownership of bed nets in households per school where children attend education (Fig. 2).

**Table 2:** Indicators of LLIN ownership, access and utilization of bed net by anyone from the household or SAC in Dara Mallo and Uba Debretsehay districts, Southern Ethiopia

Indicator	Total		Uba Debretsehay		Dara Mallo	
	n/N	% (95% CI)	n/N	% (95% CI)	n/N	% (95% CI)
Indicator I	436/2261	19.3 (17.7–21.0)	318/1419	22.4 (20.3–24.7)	118/842	14.0 (11.8–16.6)
Indicator VI	271/2261	12.0 (10.7–13.4)	179/1419	12.6 (11.0–14.5)	92/842	10.9 (8.9–13.3)
Indicator II	176/2261	7.8 (6.7–9.0)	133/1419	9.3 (7.9–11.0)	43/842	5.1 (3.8–6.9)
Indicator III	176/436	40.4 (35.8–45.2)	133/318	41.8 (36.4–47.5)	43/118	36.4 (27.9–45.9)
Indicator VII	271/436	62.2 (57.4–66.7)	179/318	56.3 (50.6–61.8)	92/118	78.0 (69.2–84.9)
Indicator IV	45/436	10.3 (7.7–13.7)	21/318	6.6 (4.2–10.1)	9/118	7.6 (3.8–14.4)
Indicator V	30/45	66.7 (50.9–79.5)	21/35	60 (42.2–75.6)	9/10	90.0 (54.1–99.5)

In univarite multilevel logistic regression analysis, the ownership of bed nets was affected by characteristics related to place of residence, household head occupation, maternal (caretaker) occupation, household head education, maternal (caretaker) education, maternal (caretaker) education level and socio-economic status. SAC households living in semi-urban area, literate educational status of the household head, literate educational status of mother (caretaker), education level of mother (caretaker) higher than grade 6 and being employee occupational status of the mother (caretaker), being employee or other occupational status of the household head and being in the 2nd or 4th quartile of the household wealth index positively and statistically significantly affected ownership of LLINs by SAC households. However, after fitting the model to multivariable multilevel logistic regression, none of the above factors significantly influenced ownership of LLINs but being in semi-urban area (AOR = 2.6; 95% CI 1.0–6.9) and employee as occupation of the household head (AOR = 1.8; 95% CI 0.9–3.7) marginally significantly affected ownership of LLINs by SAC households. The crude and adjusted ORs and the corresponding 95% CI for each of variables were presented in Table 3.

**Table 3:** Multilevel univariable and multivariable logistic regression analysis of factors affecting LLIN ownership of SAC households, 2019

Factor	Value categories	Bed net owned		COR (95% CI)	AOR (95% CI)
		No (%)	Yes (%)		
Place of residence**	Rural	1619 (84.0)	309 (16.0)	1	1
	Semi-urban	121 (65.4)	64 (34.6)	2.6 (1.–12.4)	2.6 (1.0–6.9)
	Urban	85 (57.4)	63 (42.6)	2.3 (0.7–7.7)	1.4 (0.5–4.4)
Residence house is	Private	1763 (81.3)	405 (18.7)	1	
	Not private	62 (66.7)	31 (33.3)	1.2 (0.7–2.0)	
Gender of household head	Male	1701 (80.8)	403 (19.2)	1.2 (0.7–1.9)	
	Female	125 (79.1)	33 (20.9)	1	
Age of household head	≤ 34			1	
	35–49			1 (0.76–1.32)	
	≥ 50			1 (0.6–1.5)	
Occupation of household head*	Farmer	1563 (84.4)	289 (15.6)	1	1
	Civil servant	52 (51.9)	76 (48.1)	2.6 (1.6–4.0)	1.8 (0.9–3.7)
	Merchant	81 (70.4)	34 (29.6)	1.6 (1.0–2.7)	1.6 (0.8–1.6)
	Housewife	56 (77.8)	16 (22.2)	1.2 (0.6–2.4)	1.9 (0.6–5.6)
	Daily laborer	25 (80.6)	6 (19.4)	0.7 (0.2–1.8)	0.9 (0.2–4.0)
	Others	18 (54.5)	15 (45.5)	2.5 (1.1–5.5)	2.7 (0.9–7.9)
Educational status of household head*	Illiterate	1075 (84.9)	188 (15.1)	1	1
	Literate	768 (75.6)	248 (24.4)	1.6 (1.2–2.1)	0.7 (0.4–1.4)
Age of child’s mother	≤ 34	1188 (80.7)	284 (19.3)	1	
	35–49	607 (80.8)	144 (19.2)	1.0 (0.7–1.2)	
	≥ 50	30 (79.0)	8 (21.0)	0.9 (0.4–2.0)	
Educational status of mother*	Illiterate	1379 (84.1)	260 (15.9)	1	
	Literate	446 (71.7)	176 (28.3)	1.8 (1.3–2.4)	
Education level of mother*	< Grade 7	268 (78.0)	76 (22.1)	1	1
	≥ Grade 7	138 (58.7)	97 (41.3)	1.7 (1.1–2.6)	1.3 (0.8–2.1)
Occupation of mother*	Housewife	1526 (81.7)	341 (18.3)	1	1
	Farmer	163 (86.2)	26 (13.8)	0.7 (0.3–1.3)	0.6 (0.1–2.9)
	Merchant	68 (76.4)	21 (23.6)	0.7 (0.4–1.3)	0.7 (0.3–1.5)
	Employee	47 (56.6)	36 (43.4)	2.0 (1.1–3.5)	1.2 (0.6–2.3)
	Others	21 (63.6)	12 (36.6)	1.4 (0.6–3.0)	0.9 (0.3–3.0)
Presence of pregnant mother	No	1608 (81.0)	377 (19.0)	1	
	Yes	217 (78.6)	59 (21.4)	1.2 (0.8–1.8)	
Under-fives in household	Not present	622 (81.4)	142 (18.6)	1	
	Present	1203 (80.4)	294 (19.6)	1.1 (0.8–1.4)	
Stagnant water around home	Not present	1685 (81.0)	394 (19.0)	1	
	Present	140 (76.9)	42 (23.1)	1.4 (0.8–2.3)	
Wealth index in quartile*	First	731 (85.7)	122 (14.3)	1	1
	Second	203 (71.2)	81 (28.5)	1.6 (1.1–2.3)	1.2 (0.6–2.3)
	Third	407 (72.7)	153 (27.3)	1.3 (0.9–1.8)	1.0 (0.6–1.8)
	Fourth	484 (85.8)	80 (14.2)	1.6 (1.2–2.2)	1.3 (0.8–2.2)
Altitude of residence metres above sea level**	≤ 1100	623 (73.8)	221 (26.2)	1	1
	(1100–1250)	626 (83.2)	126 (16.8)	0.6 (0.4–0.9)	
	> 1250	575 (86.6)	89 (13.4)	0.5 (0.2–1.0)	

*Statistically significant at univariable analysis* and multivariable analysis**

### LLINs utilization

**Fig. 3:**
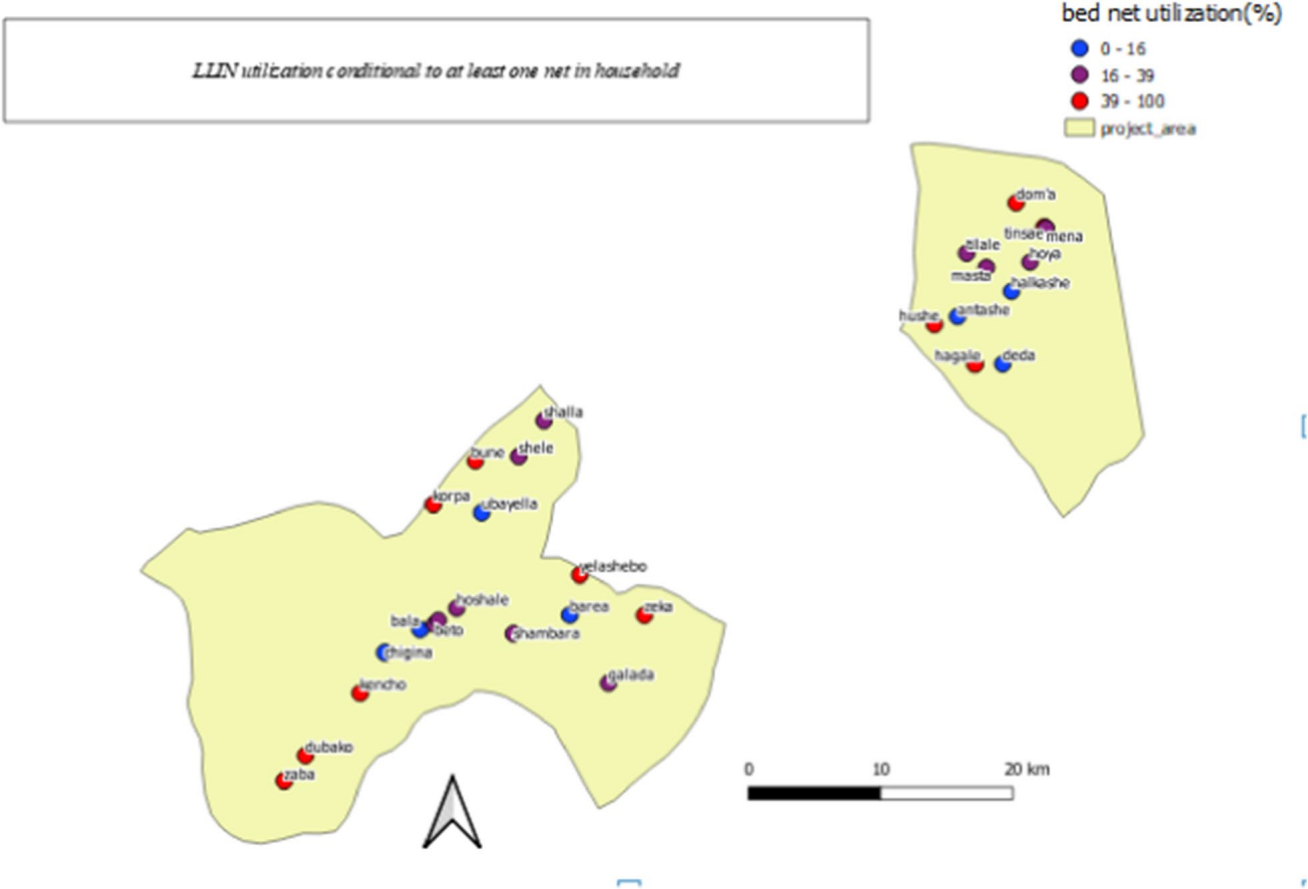
Percentage of LLIN utilization conditional to at least one LLIN in households of SAC per schools where participated children attend their education

The percentage of any person passing the previous night under a bed net from the total participating households and households conditional to bed net ownership was 12.0% (95% CI 10.7–13.4%) and 62.2% (95% CI 57.4–66.7%) respectively. About 40.3% (95% CI 35.8–45.1%) of participating SAC in the households owned at least one bed nets and 7.8% (6.7–10.0%) of all participating SAC passed the previous night under a bed net irrespective of bed net ownership. The percentage of bed net utilization among SAC conditional to adequate access of bed net in their household was 66.7% (95% CI 50.9–79.5%). The frequency, percentage and 95% CI of bed net use to prevent malaria and other indicators per the study districts was depicted in Table 2 while percentage of bed net utilization conditional to at least one bed net in the household per study schools was depicted (Fig. 3).

Passing the previous night under a bed net by SAC in univariable multilevel logistic regression analysis was affected by place of residence (COR = 4.1; 95% CI 1.5–12.4), employee being occupation of the child mother (caretaker) (COR = 3.4; 95% CI 1.4–8.1), education level above grade 6 for the child mother (caretaker) (COR = 3.9; 95% CI 1.8–9.4) and household size to bed net ratio less than or equal to 2 (COR = 4.0; 95% CI 1.8–9.8). However, after fitting the model to multivariable multilevel logistic regression, household size to bed net ratio less than or equal to 2 (AOR = 20.7; 95% CI 4.7–132.5) and child mother education level above grade 6 (caretaker) (AOR = 3.4; 95% CI 1.3–9.3) remained statistically significant. The crude and adjusted ORs and the corresponding 95% CI for each of variables assessed for bed net utilization of SAC was presented in Table 4.

**Table 4:** Univariable and multivariable logistic regression analysis LLINs utilization by SAC conditional to its ownership, 2019

Factor	Value categories	Bed net utilized		COR (95% CI)	AOR (95% CI)
		No (%)	Yes (%)		
Place of residence**	Rural	187 (60.5)	122 (39.5)	1	1
	Semi-urban	24 (37.5)	40 (62.5)	4.1 (1.5–12.4)	2.6 (0.7–11.5)
	Urban	49 (77.8)	14 (22.2)	2.3 (0.7–7.7)	0.1 (0.01–0.4)
Residence house	Private	242 (59.8)	163 (40.2)	1	
	Not private	18 (58.1)	13 (41.9)	1.2 (0.7–2.0)	
Gender of SAC	Female	133 (58.1)	96 (41.9)	1	
	male	127 (61.4)	80 (38.6)	1.0 (0.6–1.5)	
Age of the SAC	7–9	200 (57.3)	149 (42.6)	1	1
	10–14	60 (69.0)	27 (31.0)	0.8 (0.6–1.1)	
Gender of household head	Male	238 (59.1)	165 (40.9)	1.2 (0.7–1.9)	
	Female	22 (66.7)	11 (33.3)	1	
Age of household head	≤ 34	77 (68.1)	36 (31.9)	1	
	35–49	161 (57.3)	120 (42.7)	1.4 (0.8–2.4)	
	≥ 50	22 (52.4)	20 (47.6)	1.4 (0.6–3.3)	
Occupation of household head	Farmer	174 (60.2)	115 (39.8)	0.6 (0.3–1.3)	
	Civil servant	40 (52.6)	36 (47.4)	1	
	Merchant	23 (67.6)	11 (32.3)	0.4 (0.1–1.1)	
	Others	23 (62.2)	14 (37.8)	0.6 (0.2–1.6)	
Educational status of household head	Illiterate	112 (59.6)	76 (40.4)	1	
	Literate	148 (59.7)	100 (40.3)	1.1 (0.7–1.9)	
Age of child’s mother	≤ 34	173 (60.9)	111 (39.1)	1	
	35–49	83 (57.6)	61 (42.4)	1.0 (0.6–1.7)	
	≥ 50	4 (50.0)	4 (50)	1.4 (0.2–8.2)	
Educational status of mother	Illiterate	157 (60.4)	103 (39.6)	1	
	Literate	103 (58.5)	73 (41.5)	1.4 (0.8–2.4)	
Education level of mother**	< Grade 7	54 (71.1)	22 (28.9)	1	1
	≥ Grade 7	47 (48.1)	50 (51.1)	3.9 (1.8–9.4)	3.4 (1.3–9.3)
Occupation of mother*	Housewife	203 (59.5)	138 (40.5)	1	1
	Employee	14 (38.9)	22 (61.1)	3.4 (1.4–8.1)	1.9 (0.6–6.7)
	Others	43 (72.9)	16 (27.1)	0.8 (0.4–1.9)	0.3 (0.1–0.9)
Presence of pregnant mother	No	229 (60.7)	148 (39.3)	1	1
	Yes	31 (52.5)	28 (47.5)	0.7 (0.3–1.6)	
Under-fives in the household	Not present	83 (58.5)	59 (41.5)	1	
	Present	177 (60.2)	117 (39.8)	1.1 (0.7–1.9)	
Stagnant water around home	Not present	234 (59.4)	160 (36.6)	1	
	Present	26 (61.9)	16 (38.1)	1.4 (0.5–3.9)	
Knowledge of mother or care taker as only mosquito bit transmits malaria					
	No	238 (62.3)	144 (37.7)	1	
	Yes	22 (40.7)	32 (59.3)	1.2 (0.6–2.2)	
IRS last 12 months	No	107 (55.7)	85 (44.3)	1	
	Yes	153 (62.7)	91 (37.3)	0.7 (0.5–1.1)	
IRS < 4 months	Yes	85 (67.5)	41 (32.5)	1	1
	No	68 (57.6)	50 (42.4)	1.7 (0.7–4.3)	
Altitude of residence metres asl	≤ 1100	139 (62.9)	82 (37.1)	1	1
	(1100–1250)	77 (61.1)	49 (38.9)	0.9 (0.4–2.1)	
	> 1250	44 (49.4)	45 (50.6)	0.5 (0.1–2.1)	
Wealth index in quartile	First	96 (60.8)	62 (39.2)	1	
	Second	40 (61.5)	25 (38.5)	1.1 (0.5–2.2)	
	Third	59 (61.5)	37 (38.5)	0.9 (0.5–1.7)	
	Fourth	65 (55.6)	52 (44.4)	1.6 (0.9–2.9)	
Household size to bed net ratio**	> 2	245 (62.7)	146 (37.3)	1	1
	≤ 2	15 (33.3)	30 (66.7)	4.0 (1.8–9.0)	20.7 (4.7–132.5)

Statistically significant at univariable analysis* and multivariable variable analysis**

## Discussion

In this survey, ownership of LLINs and sleeping under a LLIN on the night preceding the survey by SAC were assessed. The ownership of bed nets by the households where SAC were living in the study area was 19.3%. Among those owned at least one LLIN in the household, only 10.3% had adequate access to be used by all the household members. The ownership was positively affected by being resident in semi-urban area in the study area. The percentage of children that slept the previous night under a bed net was 7.8% among the total studied; 40.3% conditional to the presence of at least one bed net in the household and 66.7% among SAC who had adequate access. Bed net utilization by the SAC conditional to presence of at least one bed net in the household was positively affected by the household size to bed net ratio equal to or less than 2 in the households and child mother (caretaker) education level above grade six.

Household bed net ownership in the present study was much lower than the universal coverage target of the NMCP [30]. Access to the bed nets which is also one of the major indicators to assess effectiveness of bed net for the prevention of malaria [31] was also poor in the current study area. Ownership of bed nets was lower than coverage estimated in the most recent malaria indicator survey in Ethiopia [13] and most other studies conducted in Ethiopia [22, 25, 32, 33] except a study conducted among households of pregnant women in Shashogo district in Southern Ethiopia [23]. The finding from the present study was also lower compared to similar studies conducted outside Ethiopia, such as at national and district levels in Uganda [34–36], Madagascar [37], Ghana [38, 39], Zimbabwe [40], Equatorial Guinea [41] and in Kenya [42]. The lower ownership of bed nets in the current study area as compared to those reviewed might be difference in the length of time between the last distribution of bed nets and the data collection period as some might be damaged or lost because of different reasons. The other possible explanation for low ownership of bed net in the study area might be due to inaccessibility of the bed nets to purchase in the districts. The difference in finding from Shashogo district in Southern Ethiopia could be related to the level of endemicity of malaria transmission being higher in the current study area.

The bed net utilization by SAC conditional to owning at least one bed net in their household was about 40% in the current study which is lower than utilization by people living in malaria-endemic areas in Africa and the overall bed net utilization in Ethiopia [1]. However, their bed net utilization was improved when access to the bed net was increased in the households. In Uganda and Zimbabwe, bed net utilization was significantly influenced by density of bed nets in households [14, 40]. The lower bed net use by the children may be related to low awareness of the community regarding vulnerability of SAC for malaria and malaria prevention measures. Bed net utilizations among SAC in Tanzania [33] and in the highlands of western Kenya [43] were higher than the finding of this study. The result of this study was in agreement to the utilization of bed net by SAC in Côte d’Ivoire [44], but higher than findings revealed from implementing national school malaria survey in Kenya [45] and bed net use among SAC after universal bed net distribution campaign in Malawi [46]. The basic reason for low coverage of bed net utilization in the present study will be due to inadequate access to the bed nets. In conditions when bed net access was not sufficient for all household members, SAC were given the least priority. The higher bed net utilization in the current study as compared to children in Malawi and Kenya could be related to difference in the background malaria transmission endemicity or cultural difference in prioritizing available bed nets to be used.

One of the two factors determining bed net utilization by SAC in the study area was the household size to bed net ratio. In conditions where bed net was not adequate to the household members, bed net utilization was determined by who is given priority from the household. A qualitative study conducted in Kutcha district near the current study area shown us that SAC were given the last priority to utilize bed nets among the household members. Pregnant women, children age less than 5 years or the household head mainly the fathers were given priority than SAC. In Malawi and Uganda SAC less likely used bed net as compared to the other population segments [35, 46]. Children age less than 5 years and pregnant women, well known vulnerable people to malaria, were more benefited from bed net utilization for the prevention of malaria. However, the insignificant association between presence of pregnant mother and under five children in the household and bed net utilization in the current study might be related to absence of bed nets to be distributed to children age less than 5 years during immunization and ANC follow-up of pregnant women from the health facilities. This was witnessed by absence of be nets to demonstrate participants in the trial mentioned in “Methods” section of this manuscript.

Bed net utilization was also influenced by economic status of population since this would provide opportunity to purchase nets in areas where there was access, as revealed by the national malaria indicator survey in SSA [47]. The finding of the present study was in contrast to this where socio-economic status of the household had no statistically significant impact on the utilization of bed nets by SAC. Lack of insignificant statistical correlation between wealth index and bed net utilization in the present study might be further explained by lack of access to purchase bed nets in their vicinity. The other factor that influenced bed net utilization in the current study was the level of maternal (caretaker) education level. This could be due to good awareness of the mothers or caretakers with higher education level regarding prevention measures of malaria.

Note should be taken of the following limitations associated with this study. The first limitation was involving only school enrolled SAC to this study as the situation could be different for school non-enrolled children. The second limitation was the use of cross-sectional study as it is not strong in generating evidence for cause and effect relationship. The strengths of this study were that the research was undertaken in a hard-to-reach and underexplored area, and had good power of the study.

## Conclusions

Ownership of bed nets, access to adequate numbers of bed nets in households of SAC and their utilization was lower than the target set to achieve universal coverage of bed nets to control malaria. It is important to monitor replacement needs of households, with main emphasis being given to households in urban area and rural areas as compared to semi-urban area. Utilization of bed nets conditional to the presence of at least one net in the household was affected by maternal education level and the density of bed nets in the household. Malaria prevention education to correct these barriers and increasing awareness about the benefit of effective and consistent utilization of insecticide treated bed nets should be given to children and mothers with low educational level to realize the elimination of malaria.

## Data Availability

The datasets used and/or analysed during the current study will be available from the corresponding author on reasonable request.

## Abbreviations

AIC: Akaike Information Criterion
AOR: Adjusted odds ratio
CI: Confidence interval
COR: Crude odds ratio
DHS: Demographic and Health Surveys
GPS: Global positioning system
IRB: Institutional Research Ethics review Board
IRS: Indoor Residual Spray
LLINs: Long-lasting insecticide-treated Nets
NMCP: National Malaria Control Programme
ODK: Open Data Kit
OR: Odds ratio
QGIS: Quantum Geographical Information System
SAC: School-aged children
SD: Standard deviation
SSA: Sub-Saharan Africa
